# Beinaglutide showed significant weight‐loss benefit and effective glycaemic control for the treatment of type 2 diabetes in a real‐world setting: a 3‐month, multicentre, observational, retrospective, open‐label study

**DOI:** 10.1002/osp4.342

**Published:** 2019-06-17

**Authors:** Y. L. Zhang, C. Zhou, X. F. Li, M. N. Yang, L. Tao, X. Y. Zheng, Y. S. Jia

**Affiliations:** ^1^ Department of Endocrinology Qianan Yanshan Hospital Hebei China; ^2^ Department of Endocrinology Qinhuangdao Hospital of Traditional Chinese Medicine Qinhuangdao Hebei China; ^3^ North China University of Science and Technology Tangshan Hebei China

**Keywords:** Beinaglutide, GLP‐1, obesity, T2DM, weight loss

## Abstract

**Aims:**

The purpose of this study was to examine the effectiveness of beinaglutide on body weight, glycated haemoglobin (HbA_1c_), blood pressure and lipid profiles in patients with type 2 diabetes mellitus (T2DM) in a real‐world setting in China.

**Materials and methods:**

This was a multicentre, observational, retrospective, open‐label study conducted in China. Data were collected from T2DM patients who started treatment with beinaglutide between 2017 and 2018.

**Results:**

A total of 314 patients were included in the study. After 3 months of treatment with beinaglutide, there were significant reductions in body weight (−10.05 kg [95% confidence interval −9.29 to −10.80]), HbA_1c_ (−2.87% [−2.62 to −3.11]), 2‐h postprandial plasma glucose (−5.46 mmol L^−1^ [−4.96 to −5.95]) and fasting plasma glucose (−3.04 mmol L^−1^ [−2.78 to −3.31]) (all *p* < 0.0001). In addition, 84.96% and 72.18% of the patients achieved weight loss of ≥5% and ≥10%, respectively. Subgroup analyses showed that weight loss was significantly greater in patients with ≥28 kg m^−2^ of baseline body mass index and 0.60 mg of beinaglutide doses (*p* = 0.007 and *p* < 0.0001, respectively). HbA_1c_ reductions were significantly greater in patients with ≥9.0% baseline HbA_1c_ and in those administered 0.40–0.48 mg of beinaglutide doses (all *p* < 0.0001). Weight loss at 3 months was positively correlated with baseline BMI and the dose of beinaglutide. Positive determinants for HbA_1c_ reduction after 3 months were baseline HbA_1c_ and the dose of beinaglutide.

**Conclusions:**

These observational results confirmed the benefits of beinaglutide in weight loss and glycaemic control and support the use of beinaglutide as an effective treatment for T2DM.

## Introduction

China is the country with the largest number of diabetic patients in the world 1. At present, the prevalence of diabetes in Chinese adults is extremely alarming, reaching as high as 11.6% 2. The total number of diabetic patients is estimated to exceed 100 million 3. Of these patients, only 25.8% received treatment for diabetes, and only 39.7% of treated patients had adequate glycaemic control 4. As one of the many drugs available for treating type 2 diabetes mellitus (T2DM), glucagon‐like peptide‐1 receptor agonists (GLP‐1RAs) have received increasing attention owing to their comprehensive effects on both glycaemic control and weight loss with low hypoglycaemia incidence.

Beinaglutide (formerly known as benaglutide), a GLP‐1RA, is recombinant human glucagon‐like peptide‐1 and shares 100% homology with human GLP‐1(7‐36) 5. The drug was approved by the China Food and Drug Administration for the treatment of T2DM in December 2016. Beinaglutide is one of the GLP‐1RAs recommended for the treatment of T2DM by the Chinese guideline for the prevention and treatment of type 2 diabetes (2017 edition) 6. The efficacy and safety of beinaglutide have been assessed in randomized controlled trials (RCTs) in China 7, 8, 9. Similar to other GLP‐1RAs, beinaglutide was effective in lowering glycated haemoglobin (HbA_1c_), fasting plasma glucose (FPG) and postprandial plasma glucose (PPG) in patients with T2DM 7, 8. In addition, beinaglutide reduced body weight and body mass index (BMI) in T2DM patients with overweight and obesity 7, 9. These RCTs provided evidence of the effectiveness of beinaglutide in a well‐defined patient population under a strictly controlled environment, yet it is uncertain to what extent the results can be translated to real‐world clinical practice where a more general patient population is treated with the new intervention in outpatient clinics.

Real‐world studies are very important to further evaluate the performance of new drugs once they reach the market. Such studies can complement RCTs and assess their results in a more general patient population. The real‐world effects of beinaglutide have not been reported to date. Thus, the purpose of this study was to examine the effectiveness of beinaglutide on HbA_1c_, body weight, blood pressure, and lipid profiles in a real‐world setting in China. The authors hypothesized that the efficacy of beinaglutide in RCTs will also be observed in the real‐world study.

## Materials and methods

This was a multicentre, observational, retrospective, open‐label study conducted in two hospitals in Hebei Province (northern China). The GLP‐1RA beinaglutide was launched in February 2017 in China. Since then, data from T2DM patients treated with beinaglutide under routine clinical practice conditions were consecutively collected until March 2018, i.e. over a time frame of 14 months. Data were extracted from the electronic medical record system and gathered in an Excel datasheet by the researchers. Adult T2DM patients (≥18 years) were eligible for the study. The exclusion criteria were patients with type 1 diabetes mellitus and those who refused to provide informed consent. The following data were collected at baseline and/or three subsequent visits (after 1, 2 and 3 months of treatment): age, sex, diabetes duration, HbA_1c_, 2‐h PPG, FPG, body weight, BMI, waist circumference (WC), C‐peptide, heart rate, systolic blood pressure (SBP), diastolic blood pressure (DBP), triglycerides, low‐density lipoprotein cholesterol (LDL‐C), high‐density lipoprotein cholesterol (HDL‐C), anti‐hypertensive and lipid‐lowering therapies and history of previous anti‐diabetic medications. Moreover, the dosage of beinaglutide and concomitant anti‐diabetic therapies was recorded. The values of HbA_1c_, FPG, 2‐h PPG, C‐peptide, triglycerides, LDL‐C and HDL‐C were extracted from standardized laboratory test results. Body weight, WC, heart rate, SBP and DBP were measured by proficient nurses using standard equipment.

The primary study objective was to assess the effectiveness of beinaglutide in controlling glycaemia after 3 months of treatment. Secondary objectives included assessing changes in body weight, the proportions of patients with weight loss ≥ 5% and ≥10% and other clinical parameters related to diabetes after 3 months of treatment. Adverse events, hypoglycaemic events and discontinuation of beinaglutide were also tracked.

This study was approved by the local ethical committees and performed in accordance with the Declaration of Helsinki (revised in 2013).

### Statistical analysis

The Kolmogorov–Smirnov test was used to determine if values were normally distributed. Data for continuous variables were expressed as the mean (standard deviation [SD]). Data for categorical variables were expressed as percentages (%). Baseline characteristics are reported on the basis of the full analysis set (FAS), which included all patients who met the eligibility criteria and had baseline measures for HbA_1c_ or weight. Efficacy analysis included all patients who received at least one dose of beinaglutide and had at least one postbaseline measurement for HbA_1c_ or body weight. Missing values were imputed using the last observation carried forward (LOCF) method. For paired samples, the paired *t*‐test or Wilcoxon matched‐pairs signed‐rank test was used for two time points, and repeated‐measures analysis of variance or the Friedman test followed by Dunn's multiple‐comparisons test was used for multiple time points. The mean (SD), mean difference, standard error (SE) and 95% confidence interval (CI) were calculated for each time point. Subgroup analyses for paired samples were performed with the same statistical methods. The changes in HbA_1c_ and weight were assessed by analysis of covariance, adjusting for baseline measures and the dose of beinaglutide as covariates. A multivariate linear regression model was applied to identify determinants of HbA_1c_ reduction and weight loss. Independent variables included age, sex, diabetes duration, baseline HbA_1c_, BMI, SBP, triglycerides, LDL‐C, HDL‐C and the dose of beinaglutide. *p* < 0.05 was considered statistically significant (two tailed). Data were analysed using spss 23 software (IBM SPSS, USA).

Based on the data without imputation for missing values (i.e. observed data only), a sensitivity analysis was conducted to assess whether the LOCF method for handling missing data might have influenced any critical conclusions.

## Results

### Baseline characteristics of patients

**Figure 1:**
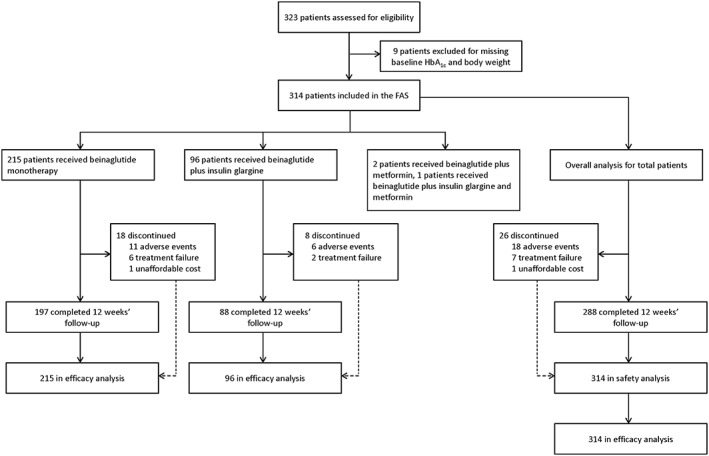
Study flow chart. FAS, full analysis set; HbA_1c_, glycated haemoglobin.

From January 2017 to March 2018, data from 323 patients treated with beinaglutide were extracted from the electronic medical record system. Of those, nine patients lacked baseline data for HbA_1c_ and body weight (unknown reasons) and were excluded from the analysis. The remaining 314 patients were included in the FAS (see flow chart in Figure 1). There were 163 (51.9%) men and 151 (48.1%) women, with an overall mean age of 47.6 (10.5) years. Most patients (60.3%) had a diabetes duration < 5 years. On average, baseline body weight was 77.94 (10.91) kg, BMI was 27.95 (4.07) kg m^−2^, HbA_1c_ was 9.05 (1.48)%, 2‐h PPG was 14.23 (3.23) mmol L^−1^ and FPG was 9.25 (1.77) mmol L^−1^. Baseline characteristics of the FAS population are summarized in Table 1. Approximately 27.7% of the FAS population were drug‐naïve patients who were newly diagnosed with T2DM. Regarding the history of previous anti‐diabetic medications before initiating beinaglutide, 30.3% of patients had been treated previously with metformin, 14.0% with repaglinide, 12.7% with acarbose, 10.8% with sulfonylurea, 8.6% with short‐acting insulin, 7.0% with basal insulin, 1.9% with DPP4 inhibitors and 1.0% with liraglutide. In addition, 48.1% of patients had received one anti‐diabetic agent, 21.0% received two anti‐diabetic agents and a small proportion (3.2%) received three anti‐diabetic agents prior to beinaglutide treatment. Regarding diabetic complications, 12.7% and 1.3% of patients presented with a personal history of coronary heart disease and brain infarction, respectively. Diabetic peripheral neuropathy, nephropathy and retinopathy were present in 40.4%, 13.7% and 2.2% of patients, respectively. A total of 10.5% of patients presented with both diabetic peripheral neuropathy and nephropathy, and a small proportion (0.1%) presented with diabetic peripheral neuropathy, nephropathy and retinopathy. Hypertension was present in 51.9% of patients, 84.0% of whom received anti‐hypertensive treatment. Dyslipidaemia was present in 33.4% of patients, 79.0% of whom received lipid‐lowering treatment.

**Table 1:** Baseline characteristics of patients before starting beinaglutide treatment

Variables	N	%	Mean (SD)
Gender	314		
Male	163	51.9	
Female	151	48.1	
Age, years	301		47.6 (10.5)
Age categories			
≤29 years	13	4.1	
30–39 years	56	17.8	
40–49 years	106	33.8	
50–59 years	82	26.1	
≥60 years	44	14.0	
Diabetes duration categories	310		
<5 years	187	60.3	
5–9 years	81	26.1	
10–14 years	33	13.6	
≥15 years	9	2.9	
HbA_1c_, %	304		9.05 (1.48)
HbA_1c_ categories			
≤7.9%	53	17.4	
8.0–8.9%	107	35.2	
≥9.0%	144	47.4	
2‐h PPG, mmol L^−1^	314		14.23 (3.23)
FPG, mmol L^−1^	303		9.25 (1.77)
C‐peptide, nmol L^−1^	110		0.95 (0.45)
Body weight, kg	314		77.94 (10.91)
BMI, kg m^−2^	310		27.78 (3.02)
BMI categories			
≤23.9 kg m^−2^	9	2.9	
24.0–27.9 kg m^−2^	172	55.5	
≥28.0 kg m^−2^	129	41.6	
WC, cm	289		95.47 (14.35)
Heart rate, bpm	123		76.05 (6.78)
SBP, mmHg	260		140.88 (18.29)
DBP, mmHg	260		86.97 (8.05)
Triglyceride, mmol L^−1^	272		2.96 (1.70)
LDL‐C, mmol L^−1^	173		1.31 (0.58)
HDL‐C, mmol L^−1^	174		3,35 (0.95)

Data are expressed as mean (SD) or percentage (%).

BMI, body mass index; bpm, beats min^−1^; DBP, diastolic blood pressure; FPG, fasting plasma glucose; HbA_1c_, glycated haemoglobin; HDL‐C, high‐density lipoprotein cholesterol; LDL‐C, low‐density lipoprotein cholesterol; PPG, postprandial glucose; SBP, systolic blood pressure; SD, standard deviation; WC, waist circumference.

### Beinaglutide administration

At baseline, 215 patients (68.5%) used beinaglutide monotherapy, 96 (30.6%) used beinaglutide in combination with insulin glargine, two (0.6%) in combination with metformin and one (0.3%) in combination with insulin glargine and metformin. At baseline, 37.6% of the patients were prescribed a daily dose of 0.2 mg, 52.3% received 0.3 mg, 5.5% received 0.4 mg, 0.5% received 0.45 mg and 4.1% received 0.6 mg of beinaglutide. Following a period of dose escalation of 1–2 weeks, prescriptions of 0.2, 0.3, 0.4, 0.42, 0.48 and 0.6 mg of beinaglutide were given to 31.2%, 5.7%, 2.1%, 11.3%, 22.7% and 27.1% of the patients, respectively. In addition, 96.0% of patients carried out supplemental lifestyle interventions.

### Clinical outcomes after 3 months of beinaglutide treatment

Clinical parameters at baseline and the 3‐month follow‐up are presented in Table 2. Compared with baseline, after 3 months of treatment, the patients showed significant reductions in body weight, HbA_1c_, 2‐h PPG, FPG, BMI, WC, heart rate, SBP, DBP, triglycerides and LDL‐C and a significant elevation in HDL‐C (all *p* < 0.0001). In subgroups, patients receiving beinaglutide monotherapy or beinaglutide in combination with insulin glargine showed similar results (*p* < 0.001). C‐peptide showed a significant reduction from baseline to 3 months in the total population of patients (*p* = 0.0016) and the patients who received monotherapy (*p* < 0.0001), but not in those receiving beinaglutide in combination with insulin glargine (*p* = 0.4464).

**Table 2:** Clinical parameters compared with baseline after 3‐month beinaglutide treatment

Variables	Total (N = 314)				Subgroup							
					Beinaglutide (N = 215)				Beinaglutide + insulin glargine (N = 96)			
	N	Baseline	After treatment	p	N	Baseline	After treatment	p	N	Baseline	After treatment	p
Body weight, kg	266	77.84 (10.70)	67.80 (10.42)	<0.0001	187	77.63 (11.19)	67.65 (10.75)	<0.0001	76	77.49 (8.28)	67.19 (8.10)	<0.0001
HbA_1c_, %	208	9.02 (1.53)	6.16 (0.87)	<0.0001	134	8.78 (1.36)	5.96 (0.93)	<0.0001	73	9.46 (1.72)	6.52 (0.63)	<0.0001
2‐h PPG, mmol L^−1^	255	14.21 (3.29)	8.75 (1.99)	<0.0001	186	14.41 (3.11)	8.98 (2.21)	<0.0001	67	13.36 (3.31)	8.12 (0.95)	<0.0001
FPG, mmol L^−1^	256	9.24 (1.79)	6.20 (1.00)	<0.0001	187	9.11 (1.65)	6.13 (1.09)	<0.0001	67	9.42 (1.76)	6.33 (0.59)	<0.0001
C‐peptide, nmol L^−1^	97	0.98 (0.45)	0.86 (0.20)	0.0016	35	1.13 (0.30)	0.94 (0.10)	<0.0001	62	0.89 (0.50)	0.82 (0.23)	0.4464
BMI, kg m^−2^	262	27.93 (4.11)	24.05 (2.95)	<0.0001	187	27.96 (4.47)	24.03 (2.90)	<0.0001	76	27.64 (2.48)	23.85 (2.33)	<0.0001
WC, cm	244	95.84 (14.63)	86.01 (12.50)	<0.0001	177	100.20 (13.96)	89.04 (12.78)	<0.0001	67	84.37 (9.17)	78.01 (7.09)	<0.0001
Heart rate, bpm	105	76.55 (6.78)	73.86 (4.27)	<0.0001	39	76.56 (7.24)	73.87 (4.12)	0.0029	66	76.55 (6.54)	73.85 (4.39)	<0.0001
SBP, mmHg	178	143.60 (19.04)	128.50 (8.78)	<0.0001	108	147.70 (16.61)	129.60 (8.37)	<0.0001	69	136.60 ( 20.45)	126.80 (9.27)	<0.0001
DBP, mmHg	178	87.97 (8.29)	81.95 (5.02)	<0.0001	108	88.92 (7.84)	81.91 (5.05)	<0.0001	69	86.55 (8.86)	82.32 (4.34)	<0.0001
Triglyceride, mmol L^−1^	188	3.09 (1.78)	1.52 (0.65)	<0.0001	115	3.18 (1.87)	1.55 (0.66)	<0.0001	73	2.95 (1.63)	1.46 (0.65)	<0.0001
LDL‐C, mmol L^−1^	148	3.38 (0.94)	1.84 (0.83)	<0.0001	79	3.44 (0.80)	2.03 (0.84)	<0.0001	69	3.31 (1.07)	1.62 (0.77)	<0.0001
HDL‐C, mmol L^−1^	147	1.31 (0.54)	2.30 (1.02)	<0.0001	78	1.18 (0.47)	1.91( 0.97)	<0.0001	69	1.46 (0.59)	2.74 (0.88)	<0.0001

Data are expressed as mean (SD).

BMI, body mass index; bpm, beats min^−1^; DBP, diastolic blood pressure; FPG, fasting plasma glucose; HbA_1c_, glycated haemoglobin; HDL‐C, high‐density lipoprotein cholesterol; LDL‐C, low‐density lipoprotein cholesterol; PPG, postprandial glucose; SBP, systolic blood pressure; SD; standard deviation; WC, waist circumference.

**Figure 2:**
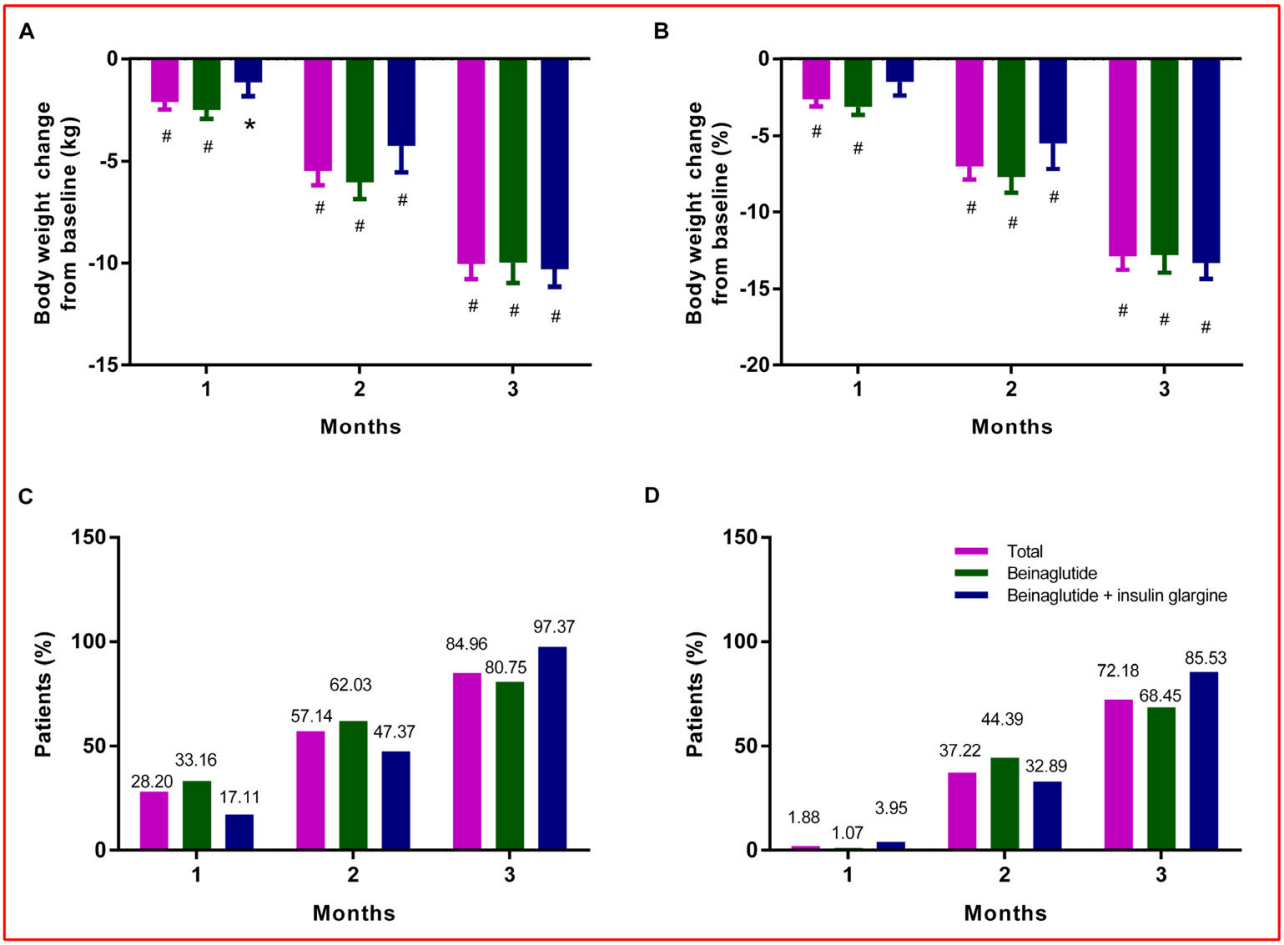
Temporal trends in body weight between baseline and 3 months. (A) Absolute change in body weight (kg). (B) Relative change in body weight (%). (C) The proportion of patients with weight loss of ≥5%. (D) The proportion of patients with weight loss of ≥10%. The bars show lower limits of 95% CIs. Significant changes from baseline are indicated (*p < 0.01, ^#^ p < 0.0001). [Correction added on 10 July 2019, after first online publication: Figure 2 has been replaced in this version.]

Temporal trends in body weight change are shown in Figure 2. Significant weight loss was observed at all time points. For the total population of patients, body weight was decreased by −10.05 kg (−9.29 to −10.80) and −12.90% (−12.02 to −13.78) after 3 months (all *p* < 0.0001). After 3 months, 84.96% and 72.18% of patients attained weight loss of ≥5% and ≥10%, respectively. Subgroup analyses showed similar results. After 3 months, body weight was decreased by −9.98 kg (−8.97 to −10.99) and −12.81% (−11.64 to −13.97) in beinaglutide monotherapy patients and by −10.30 kg (−9.43 to −11.16) and 13.33% (−12.28 to −14.38) when administered in combination with insulin glargine (all *p* < 0.0001). Furthermore, at 3 months, the proportions of patients with weight loss of ≥5% and ≥10% were 80.75% and 68.45% for monotherapy and 97.37% and 85.53% for the combination therapy with insulin glargine, respectively.

**Figure 3:**
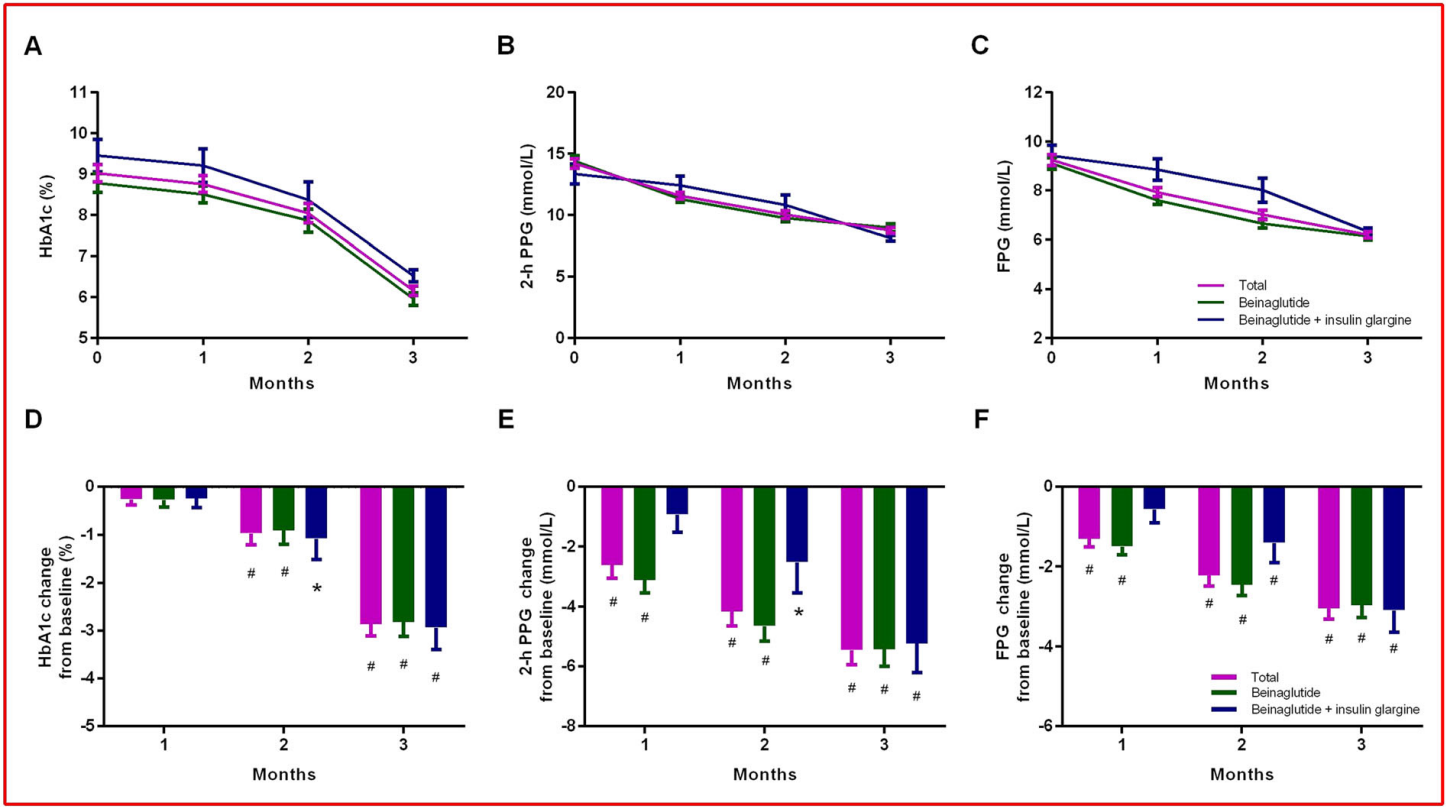
Temporal trends of glycaemic parameters between baseline and 3 months. (A) Mean values of HbA_1c_. (B) Mean values of PPG. (C) Mean values of FPG. (D) Mean change in HbA_1c_. (E) Mean change in PPG. (F) Mean change in FPG. The bars show lower limits of 95% CIs. Significant changes from baseline are indicated (*p < 0.01, ^#^ p < 0.0001). FPG, fasting plasma glucose; HbA_1c_, glycated haemoglobin; PPG, postprandial glucose.

Temporal trends in HbA_1c_, 2‐h PPG and FPG are shown in Figure 3. For the total population of patients, the average change in HbA_1c_ after 1 month was −0.26% (95% CI −0.15 to −0.38), with a further gradual reduction over time reaching −2.87% (−2.62 to −3.11) after 3 months of treatment (*p* < 0.0001). The average changes in 2‐h PPG and FPG after 3 months reached −5.46 mmol L^−1^ (−4.96 to −5.95) and −3.04 mmol L^−1^ (−2.78 to −3.31) (all *p* < 0.0001), respectively. The statistical results of the subgroups showed a similar trend. For patients receiving beinaglutide monotherapy, the average change in HbA_1c_, 2‐h PPG and FPG gradually decreased over 3 months and reached −2.83% (−2.53 to −3.12), −5.44 mmol L^−1^ (−4.88 to −5.99) and −2.97 mmol L^−1^ (−2.67 to −3.27) (all *p* < 0.0001) after 3 months of treatment, respectively. For patients receiving combination therapy with beinaglutide and insulin glargine, the average change in HbA_1c_, 2‐h PPG and FPG after 3 months reached −2.94% (−2.49 to −3.39), −5.24 mmol L^−1^ (−4.27 to −6.21) and −3.10 mmol L^−1^ (−2.55 to −3.64) (all *p* < 0.0001), respectively.

Detailed results (mean differences, standard error and 95% CI) of the temporal trends in HbA_1c_, 2‐h PPG, FPG and body weight are reported in Tables S1–S3.

### Changes in body weight and HbA_1c_ according to baseline characteristics and the dose of beinaglutide

The changes in body weight did not vary significantly by sex (*p* = 0.097), age (*p* = 0.157), diabetes duration (*p* = 0.157) or baseline HbA_1c_ categories (*p* = 0.949). The changes in HbA_1c_ also did not vary significantly by sex (*p* = 0.471), age (*p* = 0.249), diabetes duration (*p* = 0.169) or baseline BMI categories (*p* = 0.964). However, the changes in body weight were significantly greater in patients with ≥28 kg m^−2^ of baseline BMI and in patients receiving beinaglutide 0.60 mg (*p* = 0.007 and *p* < 0.0001, respectively); and the changes in HbA_1c_ were significantly greater in patients with ≥9.0% baseline HbA_1c_ and in patients receiving beinaglutide of 0.40–0.48 mg (all *p* < 0.0001) (Figure S1).

### Determinants of weight loss and HbA_1c_ reduction

The results of multiple linear regression models showed that weight loss from baseline to 3 months positively correlated with baseline BMI levels and the dose of beinaglutide, and the HbA_1c_ reduction from baseline to 3 months positively correlated with baseline HbA_1c_ levels and the dose of beinaglutide (Table S4 and S5).

### Adverse events, hypoglycaemic events and discontinuation

Among patients in the FAS, the most common adverse events were gastrointestinal symptoms, including nausea (51.0%) and vomiting (18.2%). Other reported adverse events were dizziness (17.2%) and headache (8.3%). These adverse events mainly occurred in the first month after initiation of treatment, and most were mild to moderate. A total of 14 symptomatic hypoglycaemic events were recorded within the 3 months prior to beinaglutide treatment, but no such events were reported after beinaglutide treatment. A total of 26 patients (8.3%) discontinued beinaglutide therapy within 3 months owing to gastrointestinal side effects (5.7%), treatment failure (2.2%) or unaffordable cost (0.3%).

Sensitivity analysis performed on the completer's data without LOCF imputation was generally consistent with the conclusions of the aforementioned study results (data not shown).

## Discussion

The present study is the first multicentre, observational, retrospective, open‐label analysis of beinaglutide effectiveness in T2DM patients, mostly with overweight and obesity, in China. These data demonstrated a mean weight loss of 10.05 kg and a mean reduction of 2.87% in HbA_1c_ after 3 months of treatment. These observations confirmed the effectiveness of beinaglutide in a real‐world setting.

The patients included in the present study represented a general T2DM population in a real‐world setting in China. Baseline data indicated that beinaglutide therapy was commonly prescribed for patients with overweight and obesity who had a relatively short disease duration. Notably, 27.7% of the patients were newly diagnosed with T2DM, indicating that beinaglutide was generally accepted as a second‐line treatment.

For the Chinese people, a BMI between 24.0 and 27.9 kg m^−2^ is defined as overweight, and a BMI ≥ 28.0 kg m^−2^ is categorized as obese 10. In the present study, 56.5% of patients were overweight, 41.2% were obese and only 2.3% were normal weight. When analysing the weight‐loss effect of beinaglutide on the basis of baseline BMI, patients with overweight and obesity experienced greater weight‐loss effects. This BMI‐dependent effect was similar to that in a previous study of liraglutide 11, 12. Data for normal weight Chinese patients were only available for six individuals, making estimates for this subgroup unreliable. Similar to other studies of GLP‐1RAs 13, 14, the weight‐loss effect of beinaglutide was dose dependent, with the highest dose (0.6 mg) providing the greatest weight loss. In addition, based on our results, sex, age and disease duration had no impact on drug efficacy with regard to either glycaemic control or weight loss, which was consistent with previous studies of GLP‐1RAs 11, 15.

The mean reduction in HbA_1c_ in this study was higher than mean reductions reported in the previous RCTs of beinaglutide, in which mean reductions from baseline between 0.7% and 1.2% have been reported 7, 9. This result was very interesting because HbA_1c_ reductions in real‐world groups have usually been smaller than those in RCTs according to Edelman and Polonsky's study 16. Previous studies have reported that baseline HbA_1c_ levels might predict an early response to GLP‐1RAs in terms of HbA_1c_ reductions 15, 17. In the present study, HbA_1c_ reduction was positively correlated with baseline HbA_1c_ levels; that is, i.e. patients with higher baseline HbA_1c_ levels had a greater HbA_1c_ reduction after 3 months of treatment. Compared with previous RCTs of beinaglutide (mean baseline HbA_1c_ between 7.97% and 8.05%) 7, 9, the higher baseline HbA_1c_ levels (9.02%) in this study may be an important reason for the increased efficacy observed with beinaglutide treatment.

This discrepancy might also be explained by a change in the formulation of beinaglutide. In previous RCTs, beinaglutide lyophilized powder was used for injection; that is, i.e. the powder had to be dissolved and then subcutaneously injected with a disposable sterile syringe 7, 8, 9. This formulation increased the difficulty for patients to use the drug, and the accuracy of the drug dose and patient compliance were affected. However, a new formulation (beinaglutide injection) was developed before entering the market. In clinical practice, patients can easily use an injection pen for subcutaneous administration, providing great convenience for patients prescribed beinaglutide. Thus, the change in the formulation may be another reason for the increased efficacy of beinaglutide treatment in the real‐world setting. In addition, the HbA_1c_ reductions were more significant in the relatively high‐dose groups (0.40–0.48 and 0.60 mg), and this finding was consistent with that of previous studies of GLP‐1RAs 18, 19.

Decreased fasting C‐peptide levels after beinaglutide monotherapy suggested potential efficacy for improving hyperinsulinaemia and β‐cell rest. As a short‐acting meal‐time GLP‐1RA, beinaglutide has a short half‐life (15 min) and duration of action (2 h) 6. Beinaglutide stimulates postprandial insulin secretion in a glucose‐dependent manner 20, but the insulinotropic effect is not maintained in the fasting state owing to rapid degradation of the drug. This outcome is beneficial because β‐cell rest serves to protect islet β‐cells against overstimulation and improve islet function 21, 22. This phenomenon of β‐cell rest was also observed in previous studies of GLP‐1 (7‐36) and exendin‐4 in the postprandial state 23, 24, 25. For patients receiving beinaglutide plus insulin glargine, there was a decrease in C‐peptide levels, but there was no significant difference, which might be due to lower baseline C‐peptide levels (i.e. poor β‐cell function) in this group.

The present study also provides new information about the benefits of beinaglutide on other health indicators in a real‐world setting. After 3 months of treatment, significant reductions in heart rate, SBP, DBP, total cholesterol and LDL‐C were observed, whereas HDL‐C increased significantly. These improvements in cardiovascular and lipid profiles were related to beinaglutide treatment and only partially contributed by concomitant anti‐hypertensive or lipid‐lowering treatments. These benefits together with weight loss implied a positive impact of beinaglutide on overall cardiovascular outcomes. However, this comprehensive beneficial efficacy was not unexpected, as indicated by previous studies of GLP‐1RAs 13, 26.

The safety characteristics of beinaglutide were also consistent with previous findings of GLP‐1RAs 27, 28, 29. Gastrointestinal adverse events were common but mostly transient. No symptomatic hypoglycaemic events were reported during the 3‐month treatment, which may be explained by the glucose‐dependent insulinotropic effect and short half‐life of beinaglutide. The main cause of beinaglutide discontinuation was adverse events (5.7%), which was similar to previous studies of GLP‐1RAs 30, 31.

The present study has significant strengths, such as extensive clinical information and minimal exclusion criteria, resulting in more fruitful data gained from a wide range of T2DM patients. As an observational and retrospective study, the lack of randomization and a control group are the main limitations of this study. In addition, the follow‐up period was limited to the first 3 months from the start of beinaglutide. Additional RCTs and real‐world studies are needed to evaluate the effectiveness and safety of long‐term beinaglutide treatment.

In summary, this study confirmed the effectiveness of beinaglutide on Chinese T2DM patients in a real‐world setting. Significant improvements were observed in body weight, HbA_1c_, blood pressure and lipid profiles after 3 months of treatment with beinaglutide. A trend of improvement in hyperinsulinaemia and β‐cell rest also emerged. Such benefits were observed despite a wide range of patient baseline characteristics. These observational results suggest that beinaglutide may be an effective treatment for T2DM, especially for patients with overweight and obesity, in clinical practice.

## Author contributions

Study design was performed by Y. Z. and Y. J. Data collection and analysis were conducted by C. Z., X. L., M. Y., L. T. and X. Z. Manuscript writing was carried out by Y. Z. and Y. J. All authors revised and approved the final version of the manuscript.

## Statement of assistance

The authors acknowledge Yale Duan, Ning Du and Guiyu Zhao (Shanghai Benemae Pharmaceutical Corporation) for statistical and editorial assistance.

## Funding

This study was supported by the Natural Science Foundation of Hebei Province (grant no. H2013209053). The English‐language editing fee and publication charge were funded by Shanghai Benemae Pharmaceutical Corporation.

## Conflicts of interest statement

The authors have declared that they have no conflicts of interest associated with this study.

## Supporting information


**Figure S1** Changes in HbA1c and body weight after 3 months of beinaglutide treatment. (A) Changes in HbA1c according to baseline HbA_1c_ category (the dose of beinaglutide as a covariate, among groups, p < 0.0001). (B) Changes in HbA1c according to the dose of beinaglutide (baseline HbA_1c_ as a covariate, among groups, p < 0.0001). (C) Changes in body weight according to baseline BMI category (the dose of beinaglutide as a covariate, among groups, p = 0.007). (D) Changes in body weight according to the dose of beinaglutide (baseline BMI as a covariate, among groups, p < 0.0001). Data are least squares means. The bars show lower limits of 95% CIs.
**Table S1** Temporal trends of clinical parameters in total patients.
**Table S2** Temporal trends of clinical parameters in patients receiving beinaglutide monotherapy.
**Table S3** Temporal trends of clinical parameters in patients receiving beinaglutide combination therapy with insulin glargine.
**Table S4** The determinants of HbA1c reduction after 3‐month beinaglutide treatment.
**Table S5** The determinants of weight loss after 3‐month beinaglutide treatment.
