# Supplementary material for: Beinaglutide showed significant weight‐loss benefit and effective glycaemic control for the treatment of type 2 diabetes in a real‐world setting: a 3‐month, multicentre, observational, retrospective, open‐label study
Source: Obes Sci Pract. 2019 Jun 17;5(4):366–75. doi: 10.1002/osp4.342 (PMC6700512; doi:10.1002/osp4.342)
Supplement: Supplementary file 1 — Figure S1 Changes in HbA1c and body weight after 3 months of beinaglutide treatment. (A) Changes in HbA1c according to baseline HbA1c category (the dose of beinaglutide as a covariate, among groups, p < 0.0001). (B) Changes in HbA1c according to the dose of beinaglutide (baseline HbA1c as a covariate, among groups, p < 0.0001). (C) Changes in body weight according to baseline BMI category (the dose of beinaglutide as a covariate, among groups, p = 0.007). (D) Changes in body weight according to the dose of beinaglutide (baseline BMI as a covariate, among groups, p < 0.0001). Data are least squares means. The bars show lower limits of 95% CIs. Table S1 Temporal trends of clinical parameters in total patients. Table S2 Temporal trends of clinical parameters in patients receiving beinaglutide monotherapy. Table S3 Temporal trends of clinical parameters in patients receiving beinaglutide combination therapy with insulin glargine. Table S4 The determinants of HbA1c reduction after 3‐month beinaglutide treatment. Table S5 The determinants of weight loss after 3‐month beinaglutide treatment. [file OSP4-5-366-s001.docx]

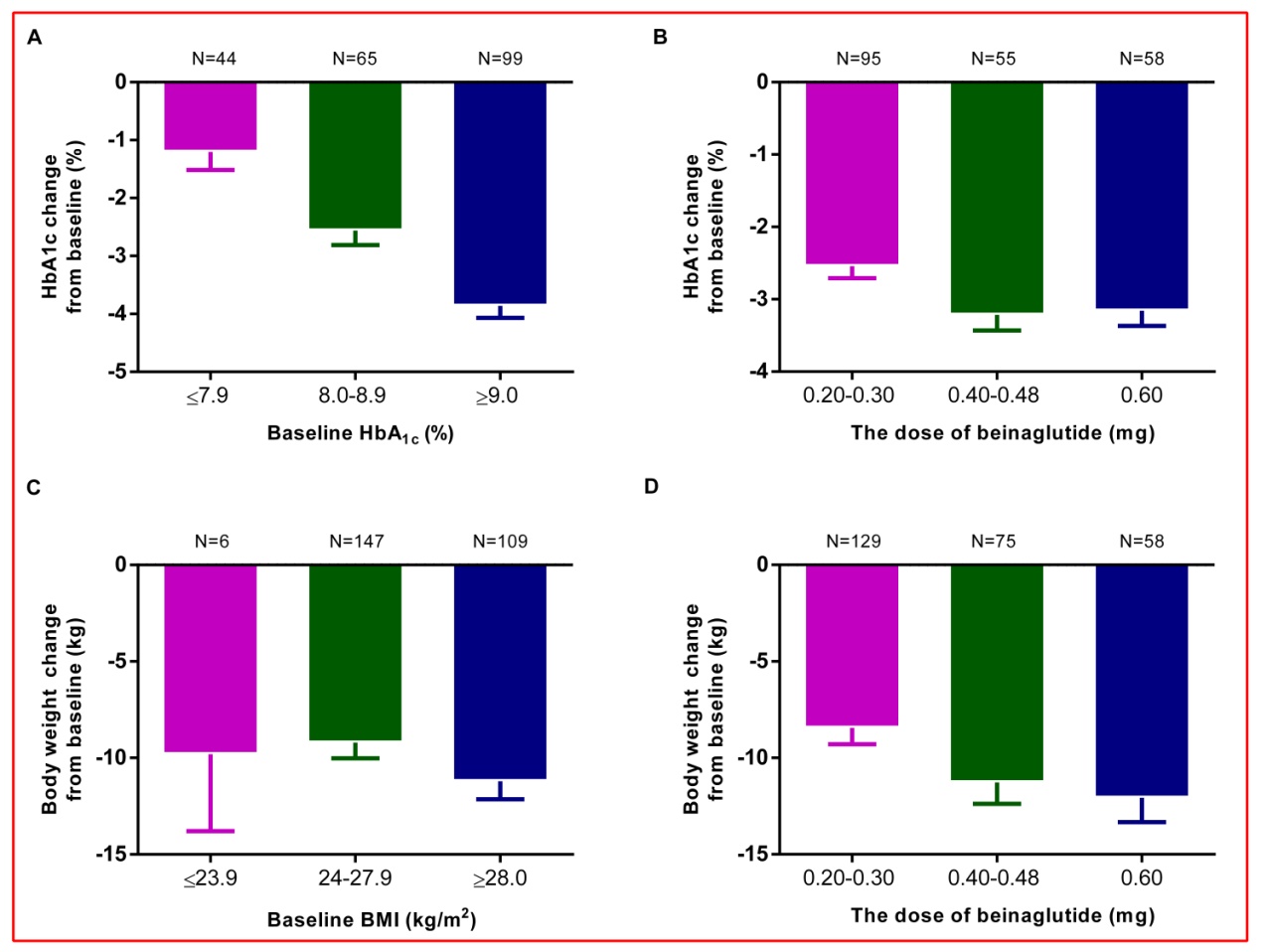


**Figure S1** Changes in HbA1c and body weight after 3 months of beinaglutide treatment. (A) Changes in HbA1c according to baseline HbA_1c_ category (the dose of beinaglutide as a covariate, among groups, *p* < 0.0001). (B) Changes in HbA1c according to the dose of beinaglutide (baseline HbA_1c_ as a covariate, among groups, *p* < 0.0001). (C) Changes in body weight according to baseline BMI category (the dose of beinaglutide as a covariate, among groups, *p* = 0.007). (D) Changes in body weight according to the dose of beinaglutide (baseline BMI as a covariate, among groups, *p* < 0.0001). Data are least squares means. The bars show lower limits of 95% CIs.

Table S1 Temporal trends of clinical parameters in total patients.

| Variables | Months | Mean (SD) | mean difference (SE) | 95% CI of difference | *P* |
| --- | --- | --- | --- | --- | --- |
| HbA_1c_, % | 0 | 9.02 (1.53) |  |  |  |
|  | 1 | 8.76 (1.49) | -0.26 (0.05) | −0.15 to −0.38 | 0.2230 |
|  | 2 | 8.05 (1.74) | -0.98 (0.10) | −0.74 to −1.22 | ＜0.0001 |
|  | 3 | 6.16 (0.87) | -2.87 (0.10) | −2.62 to −3.11 | ＜0.0001 |
| 2-h PPG, mmol/L | 0 | 14.21 (3.29) |  |  |  |
|  | 1 | 11.58 (2.36) | -2.63 (0.17) | −2.22 to −3.04 | ＜0.0001 |
|  | 2 | 10.04 (2.45) | -4.17 (0.21) | −3.68 to −4.66 | ＜0.0001 |
|  | 3 | 8.75 (1.99) | -5.46 (0.21) | −4.96 to −5.95 | ＜0.0001 |
| FPG, mmol/L | 0 | 9.24 (1.79) |  |  |  |
|  | 1 | 7.93 (1.44) | -1.31 (0.09) | −1.10 to −1.52 | ＜0.0001 |
|  | 2 | 7.02 (1.59) | -2.22 (0.11) | −1.97 to −2.48 | ＜0.0001 |
|  | 3 | 6.20 (1.00) | -3.04 (0.11) | −2.78 to −3.31 | ＜0.0001 |
| Body weight, kg | 0 | 77.84 (10.70) |  |  |  |
|  | 1 | 75.73 (10.28) | -2.12 (0.16) | −1.75 to −2.48 | ＜0.0001 |
|  | 2 | 72.35 (10.64) | -5.50 (0.29) | −4.80 to −6.19 | ＜0.0001 |
|  | 3 | 67.80 (10.42) | -10.05 (0.32) | −9.29 to −10.80 | ＜0.0001 |
| Body weight change, % | 0 |  |  |  |  |
|  | 1 |  | -2.66 (0.19) | −2.20 to −3.11 | ＜0.0001 |
|  | 2 |  | -7.02 (0.37) | −6.15 to −7.88 | ＜0.0001 |
|  | 3 |  | -12.90 (0.37) | −12.02 to −13.78 | ＜0.0001 |

Table S2 Temporal trends of clinical parameters in patients receiving beinaglutide monotherapy.

| Variables | Months | Mean (SD) | mean difference (SE) | 95% CI of difference | *P* |
| --- | --- | --- | --- | --- | --- |
| HbA_1c_, % | 0 | 8.78 (1.36) |  |  |  |
|  | 1 | 8.51 (1.22) | -0.27 (0.06) | −0.13 to −0.42 | 0.4885 |
|  | 2 | 7.87 (1.63) | -0.92 (0.12) | −0.63 to −1.21 | ＜0.0001 |
|  | 3 | 5.96 (0.93) | -2.83 (0.12) | −2.53 to −3.12 | ＜0.0001 |
| 2-h PPG, mmol/L | 0 | 14.41 (3.11) |  |  |  |
|  | 1 | 11.30 (1.91) | -3.11 (0.18) | −2.68 to −3.55 | ＜0.0001 |
|  | 2 | 9.76 (2.02) | -4.65 (0.21) | −4.15 to −5.15 | ＜0.0001 |
|  | 3 | 8.98 (2.21) | -5.44 (0.24) | −4.88 to −5.99 | ＜0.0001 |
| FPG, mmol/L | 0 | 9.11 (1.65) |  |  |  |
|  | 1 | 7.60 (1.13) | -1.50 (0.09) | −1.30 to −1.71 | ＜0.0001 |
|  | 2 | 6.65 (1.23) | -2.45 (0.11) | −2.18 to −2.72 | ＜0.0001 |
|  | 3 | 6.13 (1.09) | -2.97 (0.13) | −2.67 to −3.27 | ＜0.0001 |
| Body weight, kg | 0 | 77.63 (11.19) |  |  |  |
|  | 1 | 75.13 (10.49) | -2.51 (0.18) | −2.08 to −2.93 | ＜0.0001 |
|  | 2 | 71.57 (10.68) | -6.06 (0.35) | −5.24 to −6.87 | ＜0.0001 |
|  | 3 | 67.65 (10.75) | -9.98 (0.43) | −8.97 to −10.99 | ＜0.0001 |
| Body weight change, % | 0 |  |  |  |  |
|  | 1 |  | -3.13 (0.22) | −2.61 to −3.65 | ＜0.0001 |
|  | 2 |  | -7.72 (0.43) | −6.71 to −8.73 | ＜0.0001 |
|  | 3 |  | -12.81 (0.49) | −11.64 to −13.97 | ＜0.0001 |

Table S3 Temporal trends of clinical parameters in patients receiving beinaglutide combination therapy with insulin glargine.

| Variables | Months | Mean (SD) | mean difference (SE) | 95% CI of difference | *P* |
| --- | --- | --- | --- | --- | --- |
| HbA_1c_, % | 0 | 9.46 (1.72) |  |  |  |
|  | 1 | 9.21 (1.80) | -0.25 (0.08) | −0.06 to −0.44 | 0.7957 |
|  | 2 | 8.37 (1.88) | -1.09 (0.18) | −0.65 to −1.53 | 0.0002 |
|  | 3 | 6.52 (0.63) | -2.94 (0.19) | −2.49 to −3.39 | ＜0.0001 |
| 2-h PPG, mmol/L | 0 | 13.36 (3.31) |  |  |  |
|  | 1 | 12.43 (3.16) | -0.93 (0.25) | −0.33 to −1.52 | 0.6110 |
|  | 2 | 10.83 (3.27) | -2.53 (0.42) | −1.52 to −3.54 | 0.0002 |
|  | 3 | 8.12 (0.95) | -5.24 (0.40) | −4.27 to −6.21 | ＜0.0001 |
| FPG, mmol/L | 0 | 9.42 (1.76) |  |  |  |
|  | 1 | 8.86 (1.80) | -0.57 (0.14) | −0.23 to −0.90 | 0.3250 |
|  | 2 | 8.02 (2.03) | -1.41 (0.21) | −0.90 to −1.91 | ＜0.0001 |
|  | 3 | 6.33 (0.59) | -3.10 (0.23) | −2.55 to −3.64 | ＜0.0001 |
| Body weight, kg | 0 | 77.49 (8.28) |  |  |  |
|  | 1 | 76.32 (8.59) | -1.17 (0.28) | −0.49 to −1.85 | 0.0003 |
|  | 2 | 73.22 (9.11) | -4.26 (0.54) | −2.96 to −5.57 | ＜0.0001 |
|  | 3 | 67.19 (8.10) | -10.30 (0.36) | −9.43 to −11.16 | ＜0.0001 |
| Body weight change, % | 0 |  |  |  |  |
|  | 1 |  | -1.53 (0.37) | −0.65 to −2.40 | 0.4455 |
|  | 2 |  | -5.50 (0.70) | −3.82 to −7.18 | ＜0.0001 |
|  | 3 |  | -13.33 (0.44) | −12.28 to −14.38 | ＜0.0001 |

Table S4 The determinants of HbA1c reduction after 3-month beinaglutide treatment.

| Multivariate linear regression model | Unstandardized Coefficients | |  | Standardized Coefficients | t | *P* |
| --- | --- | --- | --- | --- | --- | --- |
|  | B | SE |  | Beta |  |  |
| (Constant) | -5.054 | 0.719 |  |  | -7.026 | < 0.0001 |
| Gender | -0.113 | 0.123 |  | -0.38 | -0.919 | 0.360 |
| Age | -0.116 | 0.062 |  | -0.084 | -1.861 | 0.065 |
| Diabetes duration | -0.055 | 0.096 |  | -0.026 | -0.573 | 0.567 |
| Baseline HbA_1c_ | 0.839 | 0.043 |  | 0.845 | 19.687 | < 0.0001 |
| Baseline BMI | -0024 | 0.014 |  | -0.071 | -1.691 | 0.093 |
| Baseline SBP | 0.006 | 0.004 |  | 0.074 | 1.617 | 0.109 |
| Baseline triglyceride | -0.011 | 0.036 |  | -0.013 | -0.311 | 0.757 |
| Baseline LDL-C | 0.093 | 0.068 |  | 0.058 | 1.380 | 0.170 |
| Baseline HDL-C | 0.060 | 0.123 |  | 0.022 | 0.485 | 0.628 |
| The dose of beinaglutide | 0.268 | 0.074 |  | 0.165 | 3.625 | < 0.0001 |

Adjusted *R^2^* was 0.788. ANOVA, *p* < 0.0001.

HbA_1c_ = glycated haemoglobin; BMI = body mass index; SBP = systolic blood pressure; LDL-C = LDL cholesterol; HDL-C = HDL cholesterol.

Table S5 The determinants of weight loss after 3-month beinaglutide treatment.

| Multivariate linear regression model | Unstandardized Coefficients | |  | Standardized Coefficients | t | *P* |
| --- | --- | --- | --- | --- | --- | --- |
|  | B | SE |  | Beta |  |  |
| (Constant) | -5.600 | 5.070 |  |  | -1.104 | 0.273 |
| Gender | -1.075 | 0.720 |  | -0.139 | -1.492 | 0.141 |
| Age | -0.024 | 0.037 |  | -0.064 | -0.631 | 0.530 |
| Diabetes duration | 0.227 | 0.531 |  | 0.042 | 0.426 | 0.671 |
| Baseline HbA_1c_ | -0.017 | 0.263 |  | -0.006 | -0.063 | 0.950 |
| Baseline BMI | 0.778 | 0.132 |  | 0.581 | 5.882 | < 0.0001 |
| Baseline SBP | -0.020 | 0.022 |  | -0.092 | -0.888 | 0.378 |
| Baseline triglyceride | -0.130 | 0.221 |  | -0.057 | -0.587 | 0.559 |
| Baseline LDL-C | 0.318 | 0.391 |  | 0.078 | 0.812 | 0.419 |
| Baseline HDL-C | -0.757 | 0.691 |  | -0.114 | -1.096 | 0.277 |
| The dose of beinaglutide | 6.493 | 2.618 |  | 0.244 | 2.480 | 0.016 |

Adjusted *R^2^* was 0.361. ANOVA, *p* < 0.0001.

HbA_1c_ = glycated haemoglobin; BMI = body mass index; SBP = systolic blood pressure; LDL-C = LDL cholesterol; HDL-C = HDL cholesterol.
